# Di-μ-pivalato-κ^3^ *O*,*O*′:*O*′;κ^3^ *O*:*O*,*O*′-bis­[(methanol-κ*O*)bis­(2,2,6,6-tetra­methylhepta­ne-3,5-dionato)praseo­dymium(III)]

**DOI:** 10.1107/S1600536811040128

**Published:** 2011-10-12

**Authors:** Qingguo Meng, Ann M. Cross, P. Stanley May, Andrew G. Sykes, Mary T. Berry

**Affiliations:** a Department of Chemistry, University of South Dakota, 414E Clark, CL115, Vermillion, SD 57069, USA

## Abstract

In the centrosymmetric dimeric title compound, [Pr_2_(C_5_H_9_O_2_)_2_(C_11_H_19_O_2_)_4_(CH_3_OH)_2_], the two praseodymium(III) atoms are eight-coordinate and are bridged by O atoms from the two pivalate anions. Each Pr^III^ ion is further coordinated by two chelating 2,2,6,6-tetra­methyl-3,5-hepta­nedionate (thd^−^) ligands and one methanol mol­ecule. The distance between the two Pr^III^ ions is 4.273 (5) Å. Intra­molecular hydrogen bonds exists between the methanol hy­droxy group on one Pr^III^ atom and a chelating O atom of a thd^−^ ligand coordinated to the symmetry-related Pr^III^ atom.

## Related literature

For general background to 2,2,6,6-tetra­methyl-3,5-hepta­nedione-based volatile complexes involving lanthanide ions, see: Sievers *et al.* (1967 ▶). For the preparation of [Pr(thd)_3_], see: Eisentraut & Sievers (1965 ▶). For a related [*Ln*_2_(thd)_6_] dimeric structure, see: Mode & Smith (1969 ▶). For an example of adducts of [*Ln*(thd)_3_], see: Baxter *et al.* (1995 ▶). For the dimeric structure of [Pr_2_(thd)_6_], see: Erasmus & Boeyens (1970 ▶). For applications of these compounds, see: Meng *et al.* (2010 ▶).
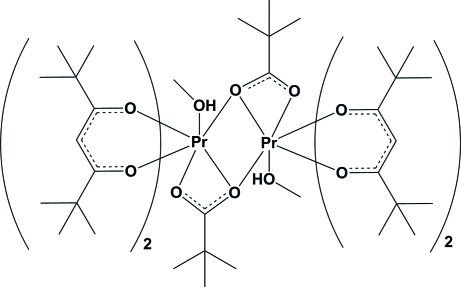


## Experimental

### 

#### Crystal data

[Pr_2_(C_5_H_9_O_2_)_2_(C_11_H_19_O_2_)_4_(CH_4_O)_2_]
*M* *_r_* = 1281.20
Monoclinic, 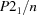

*a* = 12.6248 (14) Å
*b* = 16.5113 (18) Å
*c* = 15.5218 (17) Å
β = 94.372 (1)°
*V* = 3226.1 (6) Å^3^
*Z* = 2
Mo *K*α radiation
μ = 1.55 mm^−1^
*T* = 125 K
0.45 × 0.38 × 0.32 mm

#### Data collection

Bruker SMART APEXII diffractometer
Absorption correction: analytical (*SADABS*; Sheldrick, 1996 ▶) *T*_min_ = 0.543, *T*_max_ = 0.637
30451 measured reflections
5712 independent reflections
4618 reflections with *I* > 2σ(*I*)
*R*_int_ = 0.046

#### Refinement

*R*[*F* ^2^ > 2σ(*F* ^2^)] = 0.035
*wR*(*F* ^2^) = 0.097
*S* = 1.25
5712 reflections
329 parameters
H atoms treated by a mixture of independent and constrained refinement
Δρ_max_ = 1.54 e Å^−3^
Δρ_min_ = −0.99 e Å^−3^

### 

Data collection: *APEX2* (Bruker, 2007 ▶); cell refinement: *SAINT* (Bruker, 2007 ▶); data reduction: *SAINT*; program(s) used to solve structure: *SIR92* (Altomare *et al.*, 1994 ▶); program(s) used to refine structure: *SHELXL97* (Sheldrick, 2008 ▶); molecular graphics: *SHELXTL* (Sheldrick, 2008 ▶); software used to prepare material for publication: *WinGX* (Farrugia, 1999 ▶).

## Supplementary Material

Crystal structure: contains datablock(s) I, global. DOI: 10.1107/S1600536811040128/su2313sup1.cif

Structure factors: contains datablock(s) I. DOI: 10.1107/S1600536811040128/su2313Isup2.hkl

Additional supplementary materials:  crystallographic information; 3D view; checkCIF report

## Figures and Tables

**Table 1:** Hydrogen-bond geometry (Å, °)

*D*—H⋯*A*	*D*—H	H⋯*A*	*D*⋯*A*	*D*—H⋯*A*
O7—H1⋯O1^i^	0.71 (7)	2.04 (7)	2.741 (4)	178 (9)

Symmetry code: (i) 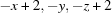.

**Fig. 1.**
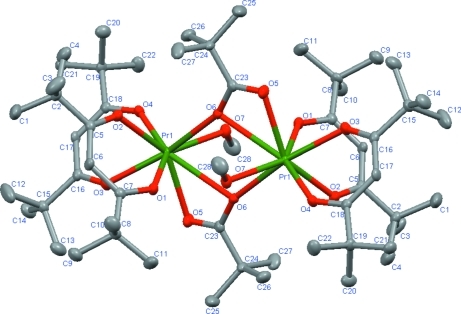
The molecular structure of the title complex, with the numbering scheme and displacement ellipsoids drawn at the 35% probability level [the H atoms have been omitted for clarity].

## supplementary crystallographic information

### Comment

2,2,6,6-tetramethyl-3,5-heptanedione (thd) lanthanide complexes have found application in many fields, such as in MOCVD, being excellent low temperature precursors. The dimeric structure of Pr_2_(thd)_6_ has been reported on previously (Erasmus & Boeyens, 1970). There each praseodymium(III) atom is surrounded by seven oxygen atoms, two of which are shared equally between the praseodymium(III) atoms.

We report herein on the crystal structure of a dimeric analog of the above mentioned complex with each Pr^III^ ion coordinating to two 2,2,6,6-tetramethyl-3,5-heptanedionate (thd^-^) ligands, one pivalate ligand, and one solvent methanol molecule. The pivalate ligand in the title compound is thought to be formed by thermal decomposition of one of a thd^-^ ligands of the precursor Pr(thd)_3_ under the current reaction conditions. The thermal decomposition mechanism is not quite clear at the moment, but it is probably due to the dissociation of the tert-butyl group from the thd^-^ ligand, followed by the rearrangement of the fragment.

The molecular structure of the title compound is illustrated in Fig. 1. It is a centrosymmetric dimeric structure where each metal is octa-coordinate, not septa as in the Pr_2_(thd)_6_ structure mentioned above. Two thd^-^ ligands chelate to each metal center in addition to a chelating pivalate anion and a methanol solvate molecule. One of the pivalate oxygen atoms, O6, bridges the two Pr^III^ atoms in a µ_2_-pivalto-O,O,O' manner; as does one of the O atoms of the thd^-^ ligands in Pr_2_(thd)_6_ (Erasmus & Boeyens, 1970). Intramolecular O-H···O hydrogen bonds involving the methanol hydroxyl group on one Pr^III^ atom with a chelating O atom of a thd^-^ ligand coordinated to the symmetry related Pr^III^ atom, stablise the molecular structure (Table 1).

Decomposition of the crystal was observed upon heating to around 340 K, before melting. So compared to Pr(thd)_3_ [Eisentraut & Sievers, 1965] the title compound cannot be considered as a good precursor for MOCVD applications.

### Experimental

Pr(thd)_3_ (where thd^-^ = 2,2,6,6-tetramethyl-3,5-heptanedionate) was prepared as described previously (Eisentraut & Sievers, 1965). For the preparation of the title compound, 0.1 g of Pr(thd)_3_ was heated to 420 K under the vacuum of 10 ^-6^ Torr for 6 h. The pivalate ligand in the title compound is thought to be formed by thermal decomposition of one of the thd^-^ ligands under the current reaction conditions. After heat-treatment, the residual product obtained was dissolved in 10 ml of methanol. The solution was stirred for 30 mins untill completely dissolved and then filtered. Green crystals, suitable for X-ray diffraction analysis, were obtained from the methanol solution, left to evaporate slowly at room temperature, after 48 h [Yield ca. 30%]. Decomposition of the crystals was observed upon heating to around 340 K, before melting.

### Refinement

The OH H atom was located in a difference Fourier map and was freely refined. The C-bound H-atoms were included in calulated positions and treated as riding atoms: C-H = 0.95 and 0.98 Å for CH and CH_3_ H-atoms, respectively, with U_iso_(H) = k × U_eq_(C), where k = 1.2 for CH H-atoms, and k = 1.5 for CH_3_ H-atoms.

### Crystal data

**Table:**

[Pr_2_(C_5_H_9_O_2_)_2_(C_11_H_19_O_2_)_4_(CH_4_O)_2_]	*F*(000) = 1336
*M**_r_* = 1281.20	*D*_x_ = 1.319 Mg m^−^^3^
Monoclinic, *P*2_1_/*n*	Mo *K*α radiation, λ = 0.71073 Å
Hall symbol: -P 2yn	Cell parameters from 9813 reflections
*a* = 12.6248 (14) Å	θ = 2.4–25.1°
*b* = 16.5113 (18) Å	µ = 1.55 mm^−^^1^
*c* = 15.5218 (17) Å	*T* = 125 K
β = 94.372 (1)°	Block, green
*V* = 3226.1 (6) Å^3^	0.45 × 0.38 × 0.32 mm
*Z* = 2	

### Data collection

**Table:**

Bruker SMART APEXII diffractometer	5712 independent reflections
Radiation source: fine-focus sealed tube	4618 reflections with *I* > 2σ(*I*)
graphite	*R*_int_ = 0.046
φ and ω scans	θ_max_ = 25.1°, θ_min_ = 1.8°
Absorption correction: analytical (*SADABS*; Sheldrick, 1996)	*h* = −15→15
*T*_min_ = 0.543, *T*_max_ = 0.637	*k* = −19→19
30451 measured reflections	*l* = −18→18

### Refinement

**Table:**

Refinement on *F*^2^	Primary atom site location: structure-invariant direct methods
Least-squares matrix: full	Secondary atom site location: difference Fourier map
*R*[*F*^2^ > 2σ(*F*^2^)] = 0.035	Hydrogen site location: inferred from neighbouring sites
*wR*(*F*^2^) = 0.097	H atoms treated by a mixture of independent and constrained refinement
*S* = 1.25	*w* = 1/[σ^2^(*F*_o_^2^) + (0.0263*P*)^2^ + 9.812*P*] where *P* = (*F*_o_^2^ + 2*F*_c_^2^)/3
5712 reflections	(Δ/σ)_max_ < 0.001
329 parameters	Δρ_max_ = 1.54 e Å^−^^3^
0 restraints	Δρ_min_ = −0.99 e Å^−^^3^

### Special details

**Table:**

Geometry. Bond distances, angles etc. have been calculated using the rounded fractional coordinates. All su's are estimated from the variances of the (full) variance-covariance matrix. The cell esds are taken into account in the estimation of distances, angles and torsion angles
Refinement. Refinement of *F*^2^ against ALL reflections. The weighted *R*-factor *wR* and goodness of fit *S* are based on *F*^2^, conventional *R*-factors *R* are based on *F*, with *F* set to zero for negative *F*^2^. The threshold expression of *F*^2^ > σ(*F*^2^) is used only for calculating *R*-factors(gt) *etc*. and is not relevant to the choice of reflections for refinement. *R*-factors based on *F*^2^ are statistically about twice as large as those based on *F*, and *R*- factors based on ALL data will be even larger.

### Fractional atomic coordinates and isotropic or equivalent isotropic displacement parameters (Å^2^)

**Table:**

	*x*	*y*	*z*	*U*_iso_*/*U*_eq_	
Pr1	0.98360 (2)	0.12656 (1)	0.97361 (1)	0.0203 (1)	
O1	1.1711 (2)	0.10441 (18)	0.9683 (2)	0.0262 (10)	
O2	1.0808 (2)	0.21145 (18)	1.07815 (19)	0.0278 (10)	
O3	1.0094 (3)	0.24206 (19)	0.8849 (2)	0.0308 (10)	
O4	0.8519 (2)	0.22213 (18)	0.9949 (2)	0.0284 (10)	
O5	0.9711 (3)	0.07544 (19)	0.8224 (2)	0.0341 (11)	
O6	0.9893 (2)	0.02507 (18)	1.08838 (18)	0.0246 (9)	
O7	0.8080 (3)	0.0510 (2)	0.9706 (2)	0.0322 (11)	
C1	1.2114 (7)	0.3914 (4)	1.0976 (4)	0.069 (3)	
C2	1.1907 (4)	0.3149 (3)	1.1468 (3)	0.0303 (16)	
C3	1.2858 (5)	0.2944 (4)	1.2099 (4)	0.064 (2)	
C4	1.0950 (5)	0.3276 (4)	1.1989 (4)	0.0503 (19)	
C5	1.1705 (4)	0.2443 (3)	1.0824 (3)	0.0258 (14)	
C6	1.2523 (4)	0.2210 (3)	1.0312 (3)	0.0310 (16)	
C7	1.2507 (4)	0.1533 (3)	0.9784 (3)	0.0275 (14)	
C8	1.3480 (4)	0.1339 (3)	0.9275 (3)	0.0324 (16)	
C9	1.3721 (5)	0.2076 (3)	0.8721 (4)	0.0462 (17)	
C10	1.4445 (4)	0.1180 (4)	0.9916 (4)	0.048 (2)	
C11	1.3275 (5)	0.0608 (4)	0.8686 (4)	0.052 (2)	
C12	1.0987 (6)	0.4229 (5)	0.8574 (4)	0.069 (3)	
C13	1.0794 (5)	0.3147 (4)	0.7452 (4)	0.0482 (19)	
C14	0.9372 (5)	0.4167 (4)	0.7546 (4)	0.0481 (19)	
C15	1.0207 (4)	0.3665 (3)	0.8080 (3)	0.0311 (14)	
C16	0.9708 (4)	0.3120 (3)	0.8746 (3)	0.0262 (12)	
C17	0.8885 (4)	0.3415 (3)	0.9219 (3)	0.0278 (16)	
C18	0.8339 (3)	0.2971 (3)	0.9793 (3)	0.0244 (12)	
C19	0.7440 (4)	0.3330 (3)	1.0288 (3)	0.0287 (16)	
C20	0.7766 (4)	0.3273 (3)	1.1259 (3)	0.0398 (17)	
C21	0.7197 (4)	0.4216 (3)	1.0063 (3)	0.0350 (17)	
C22	0.6450 (4)	0.2819 (3)	1.0081 (4)	0.0419 (19)	
C23	0.9975 (4)	0.0033 (3)	0.8346 (3)	0.0266 (12)	
C24	1.0108 (4)	−0.0528 (3)	0.7589 (3)	0.0340 (14)	
C25	1.0440 (6)	−0.0038 (4)	0.6821 (4)	0.060 (2)	
C26	1.0937 (5)	−0.1185 (3)	0.7836 (4)	0.0457 (19)	
C27	0.9012 (5)	−0.0911 (4)	0.7371 (3)	0.0490 (19)	
C28	0.7280 (5)	0.0543 (4)	0.9025 (4)	0.055 (2)	
H1	0.815 (6)	0.011 (4)	0.986 (4)	0.06 (2)*	
H1A	1.27320	0.38350	1.06400	0.1040*	
H1B	1.22520	0.43620	1.13830	0.1040*	
H1C	1.14910	0.40420	1.05840	0.1040*	
H3A	1.34880	0.28620	1.17780	0.0960*	
H3B	1.27080	0.24470	1.24140	0.0960*	
H3C	1.29870	0.33900	1.25110	0.0960*	
H4A	1.08170	0.27820	1.23130	0.0760*	
H4B	1.03260	0.34010	1.15970	0.0760*	
H4C	1.10900	0.37270	1.23930	0.0760*	
H7	1.31380	0.25440	1.03300	0.0370*	
H9A	1.38610	0.25480	0.90940	0.0690*	
H9B	1.31100	0.21870	0.83100	0.0690*	
H9C	1.43470	0.19620	0.84030	0.0690*	
H10A	1.45710	0.16530	1.02910	0.0720*	
H10B	1.50720	0.10800	0.95970	0.0720*	
H10C	1.43070	0.07050	1.02690	0.0720*	
H11A	1.26590	0.07160	0.82790	0.0780*	
H11B	1.31320	0.01320	0.90350	0.0780*	
H11C	1.39010	0.05070	0.83650	0.0780*	
H12A	1.15190	0.39080	0.89170	0.1040*	
H12B	1.06040	0.45740	0.89590	0.1040*	
H12C	1.13410	0.45680	0.81650	0.1040*	
H13A	1.02890	0.27850	0.71340	0.0720*	
H13B	1.13410	0.28240	0.77770	0.0720*	
H13C	1.11290	0.35000	0.70440	0.0720*	
H14A	0.88730	0.38030	0.72240	0.0720*	
H14B	0.97250	0.45100	0.71400	0.0720*	
H14C	0.89850	0.45070	0.79330	0.0720*	
H17	0.86870	0.39660	0.91360	0.0330*	
H20A	0.79210	0.27080	1.14150	0.0600*	
H20B	0.71850	0.34710	1.15860	0.0600*	
H20C	0.84010	0.36040	1.13970	0.0600*	
H21A	0.78340	0.45450	1.02020	0.0530*	
H21B	0.66200	0.44090	1.03980	0.0530*	
H21C	0.69830	0.42610	0.94440	0.0530*	
H22A	0.66050	0.22530	1.02300	0.0630*	
H22B	0.62320	0.28610	0.94630	0.0630*	
H22C	0.58760	0.30160	1.04170	0.0630*	
H25A	1.05250	−0.04010	0.63320	0.0900*	
H25B	1.11150	0.02370	0.69800	0.0900*	
H25C	0.98920	0.03660	0.66580	0.0900*	
H26A	1.10130	−0.15420	0.73400	0.0680*	
H26B	1.07040	−0.15040	0.83200	0.0680*	
H26C	1.16220	−0.09300	0.80070	0.0680*	
H27A	0.90490	−0.12820	0.68810	0.0730*	
H27B	0.84920	−0.04840	0.72190	0.0730*	
H27C	0.87960	−0.12110	0.78730	0.0730*	
H28A	0.66840	0.01990	0.91620	0.0820*	
H28B	0.75650	0.03500	0.84920	0.0820*	
H28C	0.70350	0.11030	0.89450	0.0820*	

### Atomic displacement parameters (Å^2^)

**Table:**

	*U*^11^	*U*^22^	*U*^33^	*U*^12^	*U*^13^	*U*^23^
Pr1	0.0231 (1)	0.0146 (1)	0.0236 (1)	−0.0010 (1)	0.0035 (1)	0.0002 (1)
O1	0.0259 (17)	0.0199 (16)	0.0330 (17)	−0.0011 (13)	0.0044 (13)	−0.0017 (13)
O2	0.0286 (18)	0.0250 (16)	0.0302 (17)	−0.0025 (14)	0.0052 (13)	−0.0039 (13)
O3	0.0314 (18)	0.0230 (17)	0.0393 (19)	0.0017 (14)	0.0111 (14)	0.0051 (14)
O4	0.0314 (18)	0.0209 (16)	0.0344 (17)	0.0014 (14)	0.0118 (14)	0.0041 (13)
O5	0.052 (2)	0.0183 (17)	0.0317 (18)	0.0037 (15)	0.0012 (15)	0.0029 (13)
O6	0.0296 (17)	0.0212 (15)	0.0233 (15)	−0.0004 (13)	0.0042 (12)	−0.0026 (12)
O7	0.0292 (19)	0.0244 (19)	0.043 (2)	−0.0015 (15)	0.0027 (15)	0.0018 (16)
C1	0.122 (7)	0.032 (3)	0.058 (4)	−0.020 (4)	0.031 (4)	−0.013 (3)
C2	0.033 (3)	0.025 (2)	0.033 (3)	−0.003 (2)	0.004 (2)	−0.007 (2)
C3	0.055 (4)	0.064 (4)	0.069 (4)	0.012 (3)	−0.012 (3)	−0.032 (4)
C4	0.048 (3)	0.044 (3)	0.060 (4)	−0.007 (3)	0.011 (3)	−0.022 (3)
C5	0.030 (3)	0.022 (2)	0.025 (2)	−0.0015 (19)	−0.0001 (18)	0.0026 (18)
C6	0.029 (3)	0.024 (2)	0.041 (3)	−0.009 (2)	0.010 (2)	−0.009 (2)
C7	0.031 (3)	0.025 (2)	0.027 (2)	0.000 (2)	0.0048 (19)	0.0040 (18)
C8	0.028 (2)	0.033 (3)	0.038 (3)	−0.004 (2)	0.015 (2)	−0.002 (2)
C9	0.050 (3)	0.044 (3)	0.047 (3)	−0.003 (3)	0.020 (3)	0.005 (3)
C10	0.035 (3)	0.051 (4)	0.060 (4)	0.007 (3)	0.012 (3)	0.006 (3)
C11	0.056 (4)	0.048 (3)	0.057 (4)	−0.012 (3)	0.031 (3)	−0.020 (3)
C12	0.081 (5)	0.076 (5)	0.050 (4)	−0.050 (4)	−0.002 (3)	0.008 (3)
C13	0.055 (4)	0.047 (3)	0.045 (3)	0.009 (3)	0.020 (3)	0.014 (3)
C14	0.058 (4)	0.041 (3)	0.047 (3)	0.009 (3)	0.015 (3)	0.017 (3)
C15	0.034 (3)	0.027 (2)	0.033 (2)	−0.002 (2)	0.007 (2)	0.003 (2)
C16	0.031 (2)	0.020 (2)	0.027 (2)	−0.0025 (19)	−0.0010 (19)	−0.0018 (18)
C17	0.035 (3)	0.015 (2)	0.034 (3)	0.0027 (19)	0.006 (2)	0.0056 (18)
C18	0.023 (2)	0.023 (2)	0.027 (2)	−0.0018 (18)	0.0002 (18)	−0.0045 (18)
C19	0.031 (3)	0.019 (2)	0.037 (3)	0.0043 (19)	0.008 (2)	0.0000 (19)
C20	0.044 (3)	0.039 (3)	0.037 (3)	0.014 (2)	0.008 (2)	0.003 (2)
C21	0.041 (3)	0.028 (3)	0.037 (3)	0.008 (2)	0.010 (2)	−0.003 (2)
C22	0.028 (3)	0.037 (3)	0.062 (4)	0.001 (2)	0.012 (2)	−0.001 (3)
C23	0.026 (2)	0.025 (2)	0.029 (2)	−0.0051 (19)	0.0027 (18)	0.0011 (19)
C24	0.059 (3)	0.023 (2)	0.021 (2)	−0.004 (2)	0.010 (2)	−0.0028 (19)
C25	0.107 (6)	0.041 (3)	0.036 (3)	−0.006 (3)	0.031 (3)	−0.001 (3)
C26	0.060 (4)	0.037 (3)	0.042 (3)	0.005 (3)	0.017 (3)	−0.009 (2)
C27	0.070 (4)	0.042 (3)	0.032 (3)	−0.014 (3)	−0.015 (3)	−0.002 (2)
C28	0.039 (3)	0.067 (4)	0.058 (4)	−0.006 (3)	−0.003 (3)	0.011 (3)

### Geometric parameters (Å, °)

**Table:**

Pr1—O1	2.403 (3)	C3—H3C	0.9800
Pr1—O2	2.408 (3)	C4—H4A	0.9800
Pr1—O3	2.389 (3)	C4—H4B	0.9800
Pr1—O4	2.334 (3)	C4—H4C	0.9800
Pr1—O5	2.488 (3)	C6—H7	0.9500
Pr1—O6	2.443 (3)	C9—H9A	0.9800
Pr1—O7	2.541 (4)	C9—H9B	0.9800
Pr1—O6^i^	2.713 (3)	C9—H9C	0.9800
O1—C7	1.289 (6)	C10—H10A	0.9800
O2—C5	1.253 (6)	C10—H10B	0.9800
O3—C16	1.259 (6)	C10—H10C	0.9800
O4—C18	1.278 (6)	C11—H11A	0.9800
O5—C23	1.247 (6)	C11—H11B	0.9800
O6—C23^i^	1.283 (5)	C11—H11C	0.9800
O7—C28	1.406 (7)	C12—H12A	0.9800
O7—H1	0.71 (7)	C12—H12B	0.9800
C1—C2	1.509 (8)	C12—H12C	0.9800
C2—C3	1.528 (8)	C13—H13A	0.9800
C2—C5	1.544 (7)	C13—H13B	0.9800
C2—C4	1.519 (8)	C13—H13C	0.9800
C5—C6	1.404 (7)	C14—H14A	0.9800
C6—C7	1.385 (7)	C14—H14B	0.9800
C7—C8	1.544 (7)	C14—H14C	0.9800
C8—C9	1.534 (7)	C17—H17	0.9500
C8—C10	1.536 (7)	C20—H20A	0.9800
C8—C11	1.524 (8)	C20—H20B	0.9800
C12—C15	1.520 (9)	C20—H20C	0.9800
C13—C15	1.530 (8)	C21—H21A	0.9800
C14—C15	1.534 (8)	C21—H21B	0.9800
C15—C16	1.541 (7)	C21—H21C	0.9800
C16—C17	1.404 (7)	C22—H22A	0.9800
C17—C18	1.378 (7)	C22—H22B	0.9800
C18—C19	1.537 (6)	C22—H22C	0.9800
C19—C21	1.530 (7)	C25—H25A	0.9800
C19—C22	1.522 (7)	C25—H25B	0.9800
C19—C20	1.535 (7)	C25—H25C	0.9800
C23—C24	1.516 (7)	C26—H26A	0.9800
C24—C26	1.536 (8)	C26—H26B	0.9800
C24—C27	1.535 (8)	C26—H26C	0.9800
C24—C25	1.526 (8)	C27—H27A	0.9800
C1—H1A	0.9800	C27—H27B	0.9800
C1—H1B	0.9800	C27—H27C	0.9800
C1—H1C	0.9800	C28—H28A	0.9800
C3—H3A	0.9800	C28—H28B	0.9800
C3—H3B	0.9800	C28—H28C	0.9800
			
O1—Pr1—O2	70.06 (10)	C2—C3—H3B	109.00
O1—Pr1—O3	85.63 (12)	C2—C3—H3C	110.00
O1—Pr1—O4	145.29 (10)	H3A—C3—H3B	109.00
O1—Pr1—O5	84.71 (11)	H3A—C3—H3C	110.00
O1—Pr1—O6	86.92 (9)	H3B—C3—H3C	109.00
O1—Pr1—O7	141.72 (11)	C2—C4—H4A	109.00
O1—Pr1—O6^i^	72.32 (9)	C2—C4—H4B	109.00
O2—Pr1—O3	80.73 (11)	C2—C4—H4C	109.00
O2—Pr1—O4	80.97 (10)	H4A—C4—H4B	109.00
O2—Pr1—O5	145.64 (11)	H4A—C4—H4C	109.00
O2—Pr1—O6	85.57 (10)	H4B—C4—H4C	110.00
O2—Pr1—O7	134.20 (10)	C5—C6—H7	117.00
O2—Pr1—O6^i^	134.79 (9)	C7—C6—H7	117.00
O3—Pr1—O4	70.84 (11)	C8—C9—H9A	110.00
O3—Pr1—O5	74.26 (10)	C8—C9—H9B	109.00
O3—Pr1—O6	165.97 (10)	C8—C9—H9C	109.00
O3—Pr1—O7	122.58 (12)	H9A—C9—H9B	109.00
O3—Pr1—O6^i^	120.39 (10)	H9A—C9—H9C	110.00
O4—Pr1—O5	111.61 (11)	H9B—C9—H9C	109.00
O4—Pr1—O6	110.01 (10)	C8—C10—H10A	109.00
O4—Pr1—O7	72.82 (10)	C8—C10—H10B	109.00
O4—Pr1—O6^i^	141.68 (9)	C8—C10—H10C	109.00
O5—Pr1—O6	116.84 (10)	H10A—C10—H10B	110.00
O5—Pr1—O7	79.84 (11)	H10A—C10—H10C	109.00
O5—Pr1—O6^i^	49.69 (9)	H10B—C10—H10C	109.00
O6—Pr1—O7	69.75 (9)	C8—C11—H11A	109.00
O6—Pr1—O6^i^	68.17 (9)	C8—C11—H11B	109.00
O6^i^—Pr1—O7	70.97 (9)	C8—C11—H11C	109.00
Pr1—O1—C7	131.4 (3)	H11A—C11—H11B	109.00
Pr1—O2—C5	134.4 (3)	H11A—C11—H11C	110.00
Pr1—O3—C16	137.5 (3)	H11B—C11—H11C	110.00
Pr1—O4—C18	138.3 (3)	C15—C12—H12A	109.00
Pr1—O5—C23	100.6 (3)	C15—C12—H12B	109.00
Pr1—O6—Pr1^i^	111.83 (10)	C15—C12—H12C	109.00
Pr1—O6—C23^i^	157.6 (3)	H12A—C12—H12B	109.00
Pr1^i^—O6—C23^i^	89.0 (3)	H12A—C12—H12C	109.00
Pr1—O7—C28	124.8 (3)	H12B—C12—H12C	110.00
Pr1—O7—H1	112 (6)	C15—C13—H13A	110.00
C28—O7—H1	111 (5)	C15—C13—H13B	109.00
C1—C2—C3	110.6 (5)	C15—C13—H13C	109.00
C3—C2—C4	107.9 (4)	H13A—C13—H13B	109.00
C3—C2—C5	109.3 (4)	H13A—C13—H13C	109.00
C4—C2—C5	110.5 (4)	H13B—C13—H13C	110.00
C1—C2—C4	109.2 (5)	C15—C14—H14A	109.00
C1—C2—C5	109.3 (4)	C15—C14—H14B	109.00
C2—C5—C6	118.8 (4)	C15—C14—H14C	109.00
O2—C5—C2	117.7 (4)	H14A—C14—H14B	109.00
O2—C5—C6	123.5 (4)	H14A—C14—H14C	109.00
C5—C6—C7	125.3 (5)	H14B—C14—H14C	110.00
O1—C7—C8	116.8 (4)	C16—C17—H17	117.00
C6—C7—C8	119.6 (4)	C18—C17—H17	117.00
O1—C7—C6	123.6 (4)	C19—C20—H20A	110.00
C7—C8—C11	111.5 (4)	C19—C20—H20B	109.00
C7—C8—C9	108.8 (4)	C19—C20—H20C	110.00
C7—C8—C10	109.1 (4)	H20A—C20—H20B	109.00
C10—C8—C11	110.1 (5)	H20A—C20—H20C	109.00
C9—C8—C11	108.9 (4)	H20B—C20—H20C	109.00
C9—C8—C10	108.5 (4)	C19—C21—H21A	109.00
C12—C15—C14	109.5 (5)	C19—C21—H21B	110.00
C12—C15—C16	107.6 (4)	C19—C21—H21C	109.00
C13—C15—C16	110.1 (4)	H21A—C21—H21B	109.00
C14—C15—C16	112.3 (4)	H21A—C21—H21C	109.00
C13—C15—C14	107.7 (4)	H21B—C21—H21C	110.00
C12—C15—C13	109.7 (5)	C19—C22—H22A	109.00
C15—C16—C17	120.3 (4)	C19—C22—H22B	109.00
O3—C16—C15	116.5 (4)	C19—C22—H22C	109.00
O3—C16—C17	123.1 (4)	H22A—C22—H22B	110.00
C16—C17—C18	125.4 (5)	H22A—C22—H22C	109.00
C17—C18—C19	122.9 (4)	H22B—C22—H22C	109.00
O4—C18—C17	123.2 (4)	C24—C25—H25A	110.00
O4—C18—C19	114.0 (4)	C24—C25—H25B	110.00
C18—C19—C22	107.9 (4)	C24—C25—H25C	109.00
C20—C19—C21	108.5 (4)	H25A—C25—H25B	110.00
C20—C19—C22	109.1 (4)	H25A—C25—H25C	109.00
C21—C19—C22	109.6 (4)	H25B—C25—H25C	109.00
C18—C19—C21	113.4 (4)	C24—C26—H26A	110.00
C18—C19—C20	108.3 (4)	C24—C26—H26B	109.00
O5—C23—O6^i^	120.3 (4)	C24—C26—H26C	110.00
O5—C23—C24	120.7 (4)	H26A—C26—H26B	109.00
O6^i^—C23—C24	119.0 (4)	H26A—C26—H26C	110.00
C23—C24—C26	110.6 (4)	H26B—C26—H26C	109.00
C23—C24—C27	105.7 (4)	C24—C27—H27A	109.00
C25—C24—C27	110.1 (4)	C24—C27—H27B	109.00
C26—C24—C27	110.5 (4)	C24—C27—H27C	110.00
C25—C24—C26	110.3 (5)	H27A—C27—H27B	109.00
C23—C24—C25	109.6 (4)	H27A—C27—H27C	109.00
C2—C1—H1A	109.00	H27B—C27—H27C	109.00
C2—C1—H1B	110.00	O7—C28—H28A	110.00
C2—C1—H1C	109.00	O7—C28—H28B	109.00
H1A—C1—H1B	110.00	O7—C28—H28C	109.00
H1A—C1—H1C	109.00	H28A—C28—H28B	109.00
H1B—C1—H1C	109.00	H28A—C28—H28C	110.00
C2—C3—H3A	109.00	H28B—C28—H28C	109.00
			
O2—Pr1—O1—C7	−35.6 (4)	O4—Pr1—O7—C28	73.0 (4)
O3—Pr1—O1—C7	46.1 (4)	O5—Pr1—O7—C28	−43.6 (4)
O4—Pr1—O1—C7	−0.4 (5)	O6—Pr1—O7—C28	−167.3 (4)
O5—Pr1—O1—C7	120.7 (4)	O6^i^—Pr1—O7—C28	−94.3 (4)
O6—Pr1—O1—C7	−122.0 (4)	Pr1—O1—C7—C8	−149.2 (3)
O7—Pr1—O1—C7	−173.1 (3)	Pr1—O1—C7—C6	30.4 (7)
O6^i^—Pr1—O1—C7	169.9 (4)	Pr1—O2—C5—C2	161.8 (3)
O1—Pr1—O2—C5	29.9 (4)	Pr1—O2—C5—C6	−17.4 (7)
O3—Pr1—O2—C5	−58.8 (4)	Pr1—O3—C16—C15	177.7 (3)
O4—Pr1—O2—C5	−130.7 (4)	Pr1—O3—C16—C17	−0.6 (8)
O5—Pr1—O2—C5	−15.4 (5)	Pr1—O4—C18—C17	15.6 (7)
O6—Pr1—O2—C5	118.2 (4)	Pr1—O4—C18—C19	−165.7 (3)
O7—Pr1—O2—C5	174.1 (4)	Pr1—O5—C23—O6^i^	7.1 (5)
O6^i^—Pr1—O2—C5	65.2 (4)	Pr1—O5—C23—C24	−175.0 (4)
O1—Pr1—O3—C16	−146.9 (5)	Pr1^i^—O6^i^—C23—O5	152.6 (5)
O2—Pr1—O3—C16	−76.4 (5)	Pr1—O6^i^—C23—C24	175.6 (4)
O4—Pr1—O3—C16	7.2 (4)	Pr1^i^—O6^i^—C23—C24	−25.4 (10)
O5—Pr1—O3—C16	127.4 (5)	Pr1—O6^i^—C23—O5	−6.4 (5)
O7—Pr1—O3—C16	60.8 (5)	C1—C2—C5—O2	−116.5 (6)
O6^i^—Pr1—O3—C16	146.6 (4)	C3—C2—C5—C6	−58.5 (6)
O1—Pr1—O4—C18	34.9 (5)	C4—C2—C5—O2	3.8 (6)
O2—Pr1—O4—C18	68.2 (4)	C3—C2—C5—O2	122.3 (5)
O3—Pr1—O4—C18	−15.1 (4)	C4—C2—C5—C6	−177.1 (5)
O5—Pr1—O4—C18	−78.6 (4)	C1—C2—C5—C6	62.7 (6)
O6—Pr1—O4—C18	150.0 (4)	O2—C5—C6—C7	−8.9 (8)
O7—Pr1—O4—C18	−149.9 (4)	C2—C5—C6—C7	171.9 (5)
O6^i^—Pr1—O4—C18	−130.2 (4)	C5—C6—C7—C8	−178.3 (4)
O1—Pr1—O5—C23	67.4 (3)	C5—C6—C7—O1	2.1 (8)
O2—Pr1—O5—C23	109.5 (3)	O1—C7—C8—C10	−118.1 (5)
O3—Pr1—O5—C23	154.4 (3)	O1—C7—C8—C11	3.7 (6)
O4—Pr1—O5—C23	−144.2 (3)	O1—C7—C8—C9	123.8 (5)
O6—Pr1—O5—C23	−16.4 (3)	C6—C7—C8—C11	−175.9 (5)
O7—Pr1—O5—C23	−77.4 (3)	C6—C7—C8—C10	62.3 (6)
O6^i^—Pr1—O5—C23	−3.8 (3)	C6—C7—C8—C9	−55.9 (6)
O1—Pr1—O6—Pr1^i^	−72.17 (11)	C13—C15—C16—O3	20.5 (6)
O2—Pr1—O6—Pr1^i^	−142.39 (11)	C12—C15—C16—C17	79.4 (6)
O4—Pr1—O6—Pr1^i^	138.90 (10)	C13—C15—C16—C17	−161.1 (5)
O5—Pr1—O6—Pr1^i^	10.32 (15)	C14—C15—C16—O3	140.5 (5)
O7—Pr1—O6—Pr1^i^	76.90 (11)	C14—C15—C16—C17	−41.2 (6)
O6^i^—Pr1—O6—Pr1^i^	0.03 (14)	C12—C15—C16—O3	−99.0 (5)
O1—Pr1—O6—C23^i^	85.1 (7)	C15—C16—C17—C18	176.1 (4)
O2—Pr1—O6—C23^i^	14.9 (7)	O3—C16—C17—C18	−5.7 (8)
O4—Pr1—O6—C23^i^	−63.8 (7)	C16—C17—C18—C19	−179.7 (4)
O5—Pr1—O6—C23^i^	167.6 (7)	C16—C17—C18—O4	−1.1 (8)
O7—Pr1—O6—C23^i^	−125.8 (8)	C17—C18—C19—C20	−119.0 (5)
O6^i^—Pr1—O6—C23^i^	157.3 (8)	C17—C18—C19—C21	1.5 (6)
O1^i^—Pr1^i^—O6—Pr1	−93.84 (12)	C17—C18—C19—C22	123.0 (5)
O2^i^—Pr1^i^—O6—Pr1	−59.01 (16)	O4—C18—C19—C21	−177.2 (4)
O3^i^—Pr1^i^—O6—Pr1	−167.60 (12)	O4—C18—C19—C22	−55.7 (5)
O4^i^—Pr1^i^—O6—Pr1	95.03 (16)	O4—C18—C19—C20	62.3 (5)
O5^i^—Pr1^i^—O6—Pr1	167.90 (17)	O6^i^—C23—C24—C26	−31.4 (6)
O6^i^—Pr1^i^—O6—Pr1	0.03 (12)	O6^i^—C23—C24—C27	88.2 (5)
O7^i^—Pr1^i^—O6—Pr1	75.15 (11)	O6^i^—C23—C24—C25	−153.2 (5)
O1—Pr1—O7—C28	−111.4 (4)	O5—C23—C24—C25	28.9 (7)
O2—Pr1—O7—C28	131.0 (4)	O5—C23—C24—C26	150.7 (5)
O3—Pr1—O7—C28	20.2 (4)	O5—C23—C24—C27	−89.7 (6)

Symmetry codes: (i) −*x*+2, −*y*, −*z*+2.

### Hydrogen-bond geometry (Å, °)

**Table:**

*D*—H···*A*	*D*—H	H···*A*	*D*···*A*	*D*—H···*A*
O7—H1···O1^i^	0.71 (7)	2.04 (7)	2.741 (4)	178 (9)

Symmetry codes: (i) −*x*+2, −*y*, −*z*+2.
